# Clinically Apparent Arterial Thrombosis in Persons with Systemic Vasculitis

**DOI:** 10.1155/2017/3572768

**Published:** 2017-06-21

**Authors:** Alexander Tsoukas, Sasha Bernatsky, Lawrence Joseph, David L. Buckeridge, Patrick Bélisle, Christian A. Pineau

**Affiliations:** ^1^Division of Rheumatology, Department of Medicine, McGill University Health Centre, Montreal, QC, Canada; ^2^Division of Clinical Epidemiology, Department of Medicine, McGill University Health Centre, Montreal, QC, Canada; ^3^McGill Clinical and Health Informatics, McGill University, Montreal, QC, Canada; ^4^Department of Epidemiology, Biostatistics and Occupational Health, McGill University, Montreal, QC, Canada; ^5^Office of Surveillance and Epidemiology, Montreal Public Health Department, Montreal, QC, Canada

## Abstract

**Objective:**

To estimate the incidence rate of clinically apparent arterial thrombotic events and associated comorbidities in patients with primary systemic vasculitis.

**Methods:**

Using large cohort administrative data from Quebec, Canada, we identified patients with vasculitis, including polyarteritis nodosa (PAN) and granulomatosis with polyangiitis (GPA). Incident acute myocardial infarctions (AMIs) and cerebrovascular accidents (CVAs) after the diagnosis of vasculitis were ascertained in the PAN and GPA group via billing and hospitalization data. These were compared to rates of a general population comparator group. The incidences of comorbidities (type 2 diabetes mellitus, dyslipidemia, and hypertension) were also collected.

**Results:**

Among the 626 patients identified with vasculitis, 19.7% had PAN, 2.9% had Kawasaki disease, 23.8% had GPA, 52.4% had GCA, and 1.3% had Takayasu arteritis. The AMI rate was substantially higher in males aged 18–44 with PAN, with rates up to 268.1 events per 10,000 patient years [95% CI 67.1–1070.2], approximately 30 times that in the age- and sex-matched control group. The CVA rate was also substantially higher, particularly in adults aged 45–65. Patients with vasculitis had elevated incidences of diabetes, dyslipidemia, and hypertension versus the general population.

**Conclusion:**

Atherothrombotic rates were elevated in patients identified as having primary systemic vasculitis. While incident rates of cardiovascular comorbidities were also increased, the substantial elevation in AMIs seen in young adults suggests a disease-specific component which requires further investigation.

## 1. Introduction

Primary systemic vasculitis encompasses a group of disorders characterized by inflammation and necrosis of blood vessels which leads to end-organ damage. Over the past two decades, treatments have significantly improved survival, resulting in increased chronicity of disease and a shift towards treating associated comorbidities. Of importance is the potentially heightened degree of atherosclerotic disease seen in several types of systemic vasculitis including giant cell arteritis [1, 2], Kawasaki's disease [3], granulomatosis with polyangiitis (GPA) [4–8], microscopic polyangiitis (MPA) [6, 7], and polyarteritis nodosa (PAN) [7, 9–13].

The low incidence of systemic vasculitis in the general population makes the creation of large prospective clinical cohorts difficult. Among these patients, investigating relatively rare outcomes such as acute myocardial infarction (AMI) and cerebrovascular accidents (CVA) poses an even greater challenge. In recent years, administrative health databases have been used to not only estimate incidence of several rheumatologic diseases but also monitor a variety of associated health outcomes. Here we estimate the incidence of clinically apparent AMI and CVA from a large administrative database in subjects with systemic vasculitis, further divided into PAN and GPA. We then evaluate the incidence of cardiovascular risk factors within these groups in the period after being diagnosed with vasculitis, compared to the incidence in the general population.

## 2. Materials and Methods

### 2.1. Data

We identified a cohort of individuals diagnosed with primary systemic vasculitis, as well as individuals specifically having GPA and PAN, from the Quebec provincial billing and hospitalization databases over the period Jan 1, 1994–Dec 31, 2003. These databases contain information on the approximately 7.5 million residents within the province of Quebec. Every physician visit record includes the date of the visit and a single diagnostic code (according to the International Classification of Diseases, ICD, version 9). Similarly, the hospitalization database contains information on all admissions, with each patient receiving a primary discharge diagnosis and up to fifteen secondary diagnoses (coded using ICD-9 during this time period).

### 2.2. Study Cohort

Adult patients required at least 1 hospital discharge diagnosis of ICD-9 code 446.0, 1, 4, 5, or 7, or at least two of these billing codes at least 8 weeks apart within a two-year period made by a rheumatologist, immunologist, neurologist, nephrologist, dermatologist, otolaryngologist, respirologist, or internist. These ICD-9 codes map to the following subgroups: PAN (446.0), Kawasaki's (446.1), GPA (446.4), GCA (446.5), and Takayasu's (446.7). With this system, there is no specific code for MPA or eosinophilic granulomatosis with polyangiitis. Subgroups PAN (ICD-9 code 446.0) and GPA (ICD-9 code 446.4) were defined by the same method. Cohort subjects were followed from the index date of diagnosis until the first of outcome event, death, or end of study.

### 2.3. General Population Comparator

A separate cohort was created as a general population comparator. This included individuals who have lived at least one year in the census metropolitan area of Montreal between 1996 and 2006 and who had been given a physician billing diagnosis of upper respiratory tract infection or flu-like symptoms in the provincial billing and hospitalization databases during this period [14]. This cohort has just under 3 million individuals and represents a general population sample of urban residents who access health care.

### 2.4. Outcome Definitions

Our two primary outcomes were new arterial thrombotic events defined by AMI (ICD-9 code 410.x) or CVA (ICD-9 code 433.x, 434.x). Each event was defined by an algorithm requiring at least one hospitalization diagnosis.

Our comorbidity assessments were defined by diagnoses of type 2 diabetes mellitus (ICD-9 code 250.x) [15], hypertension (401x–405x) [16], and dyslipidemia (272.x). For each comorbidity, diagnosis was based on at least ≥ 1 hospitalization code or ≥2 physician billing claims.

The period of assessment of these three comorbidities in patients with vasculitis began 1 year prior to their first diagnostic code and continued up to 1 year after. For the general population comparator group, the 2-year assessment period for these comorbidities was the first 2 years of available data after the index date.

In both groups, incidence rates for the outcomes of events were calculated with 95% confidence intervals, assuming a Poisson distribution.

The Quebec Commission D'Accès à l'Information allowed us to access to the administrative data (stripped of nominal identifying information). The study protocol is consistent with the principles of the Declaration of Helsinki and was approved by the McGill University Institutional Review Board.

## 3. Results

626 patients diagnosed with primary systemic vasculitis were identified within the observation period, of which 19.7% had PAN and 23.8% had GPA, 52.4% had GCA, 2.9% had Kawasaki disease, and 1.3% had Takayasu arteritis. The demographics of the PAN and GPA group are presented in Table 1.

Fifteen AMIs occurred in the 124 patients with PAN, and 5 AMIs occurred in the 152 patients with GPA. Ten CVAs occurred in patients with PAN, and 5 CVAs occurred in patients with GPA (see Table 2). In those vasculitis patients who had AMI, 45.8% occurred within the first year of follow-up, 8.5% occurred between the second and third year of follow-up, and the remainder occurred thereafter. In those vasculitis patients who had a CVA, 36.7% occurred within the first year of follow-up, 10% occurred between the second and third year of follow-up, and the remainder occurred thereafter.

In males with vasculitis, the incidence rate of AMI was substantially higher in those aged 18–44 years. In this age group, in PAN we estimated 268.1 events per 10,000 patient years [95% CI 67.1–1070.2] compared to 8.9 events per 10,000 patient years [95% CI 8.7–9.2] in the general population. There was also a higher AMI incidence in the 45–65-year-old group with PAN, compared to the general population of this age group.

In females, the incidence rate of AMI was substantially higher in those aged 18–44 years in the PAN group at 172.3 events per 10,000 patient years [95% CI 43.1–689.0] and the GPA group at 71.5 events per 10,000 patient years [95% CI 10.1–507.5], compared to 1.95 events per 10,000 patient years [95% CI 1.86–2.05] in the general population group.

The incidence rate of CVA in males aged 45–65 years was again higher in both the PAN group at 381.4 events per 10,000 patient years [95% CI 143.2–1016.3] and the GPA group at 147.2 events per 10,000 patient years [95% CI 36.8–588.5], compared to 23.6 events per 10,000 patient years [95% CI 23.0–24.1] in the general population group. While a trend was seen within the PAN over-65 age groups, again definitive conclusions could not be reached. CVA incidence in the 18–44 age group was very low.

In females aged 45–65 there was an elevation of CVA events in the PAN group at 182.8 events per 10,000 patient years [95% CI 45.7–731.1] and the GPA group at 112.4 events per 10,000 patient years [95% CI 28.1–449.7] compared to 13.2 events per 10,000 patient years [95% CI 12.8–13.6] in the general population group. In females aged over 65 years, we were unable to determine a definite difference in either outcome.

### 3.1. Comorbidity Assessment

Patients with vasculitis had elevated occurrence of diabetes, dyslipidemia, and hypertension compared to that within the age and sex specific general population strata (data not shown). Compared to the general population, clear increases in dyslipidemia were apparent in males and females aged less than 45 years with GPA and in 45–65-year-old males with PAN. As well, compared to the general population, clear increases in hypertension were observed in males of any age with PAN and GPA and in females aged 45–65 with PAN. However, comorbidities in those under 45 years of age were seen relatively rarely.

## 4. Discussion

Several factors may lead to increased arterial thrombotic events seen in vasculitis. These include systemic inflammation, localized vascular inflammation, and treatment-related effects. Endothelial dysfunction is hypothesized to be a leading pathophysiological mechanism of early atherosclerosis in vasculitis patients caused by generalized inflammation [17–20], and several studies have noted increased surrogate markers of atherosclerosis, such as carotid intima-media thickness, and raised endothelial activation markers [7, 21–24]. In addition, localized inflammation and vascular damage by the vasculitic process may contribute to aneurysm formation and vascular ectasia, which leads to vascular stenosis or rupture resulting in myocardial infarction or stroke [25–27].

Our findings are in agreement with previous studies which have demonstrated increased incidences of arterial thrombotic events in patients with systemic necrotizing vasculitis [6–8]. The increased diagnosis rates of AMI in patients under 45 years of age with PAN are most striking, with up to 268.1 events per 10,000 patient years, over 30 times higher than individuals without vasculitis. The cause of such an elevation may be multifactorial.

There has been some suggestion in past literature that PAN is associated with acute cardiovascular events [10–12, 28–39]. A study done using coronary magnetic resonance angiography in patients with small-vessel vasculitis revealed an unexpectedly high incidence of coronary ectasias and aneurysms in patients with MPA and PAN, as well as GPA, which were not seen at all in patients with RA and SLE (control groups who also suffer from chronical inflammation and are often on prolonged steroids) [13]. In vasculitis, there may be a disease-specific element which causes acute and/or chronic intrinsic vascular damage leading to atherothrombotic events, even in relatively young patients.

The strengths of our study included creation of a cohort from the entire provincial population, with follow-up over multiple years. The use of patient data from an administrative database minimized selection and recall bias that might be introduced if subjects were asked to recall their medical history by self-report, retrospectively. Inherent to all epidemiological studies using administrative data is the issue of correctly defining cases and outcomes. Validation studies using administrative databases have been done in several rheumatologic diseases, with a variety of case ascertainment algorithms which balance sensitivity and specificity for the case of interest. The use of at least two billing diagnoses and/or at least one hospital diagnosis for the disease of interest is a commonly used methodology [40]. At the time of this study, we were aware of no validation studies using administrative data for GPA or PAN. However, three validation studies for Kawasaki disease, a rare vasculitis of childhood, using a case-finding algorithm with a single ICD-9 code (446.1 on discharge diagnosis) showed a positive predictive value of 74–86% [41–43] for correctly identifying the disease. Our case definition of 1 hospitalization or 2 billing diagnoses within an 8-week period made by a specialist is comparatively stringent, as a large proportion of previous validation studies in other conditions have used single billing diagnoses, or generalist data. The incidence of PAN in our cohort was 1.65/million/year which compares favourably to prior large cohort estimates of 0.9–9.7/million/year [44, 45], as was the incidence of GPA of 2.1/million/year compared to prior figures of 0.7–11.2/million/year [46].

In addition, our outcome definitions of a single hospitalization for AMI have been previously validated with good sensitivity and specificity [47, 48]. To our knowledge, there are no codes to differentiate acute MI due to coronary arteritis, from that due to chronic atherosclerosis; however the younger age of patients with PAN suffering AMIs could suggest arteritis and not atherosclerosis as the cause. The incidence of CVA using a single hospitalization code is associated with increased specificity [49].

Similarly, the algorithms we used in our comorbidity assessments are similar to those which have been previously validated for diabetes and hypertension [15, 16]. Due to limitations in billing data, we were unable to include other comorbidities such as smoking history. In addition, as only one physician diagnostic code is used for every visit in Quebec administrative data, incomplete ascertainment of comorbidity is possible, particularly in the setting of multimorbidity. Due to vasculitis being difficult to diagnose, there may also be misclassification of some cases by specialists. Interestingly, we noted a relatively high prevalence of vasculitis in female patients, which may be due to the fact that females have more medical encounters compared to males in Canada, or possibly due to males having more severe disease and dying more rapidly of vasculitis related complications, than females. Alternatively, the sensitivity and specificity of our vasculitis case definitions may have been varied between males and females.

We noted fairly high AMI and CVA event rates in our general population comparator, particularly in the >65-year-old group. This likely derives from the fact that the general comparator group was formed by patients with upper respiratory tract infections, who are generally sicker patients prone to cardiovascular events. This is reflected in the over-65-year-old male comparator group who, for instance, had a myocardial infarction rate of 181.1/10,000 patient/years, compared to historical Canadian figures of 112.2/10,000 patient/years [50]. Although the control group may have overestimated events in older adults, it is likely a valid representation of the general population in a younger age group who may not be as prone to thrombotic events after an URTI [14]. Regardless, our choice of control group may have tended to underestimate an increased risk of AMI and CVA within vasculitis patients, compared to the general population.

In summary, in our sample, the risk of AMI and CVA appeared significantly higher in PAN and GPA, which may result from both comorbidity effects and unique disease-related processes. Additional studies defining the nature of such early cardiovascular events in this patient group are warranted.

## Figures and Tables

**Table 1:** Demographics.

	PAN^+^ *n* = 124 (14.8%)	GPA^++^ *n* = 152 (18.2%)	Control *n* = 2,868,629
*Males*	**58 (46.8%)**	**65 (42.8%)**	**1,326,132 (46.2%)**
18–44	14 (11.3%)	16 (10.5%)	867,070 (30.2%)
45–65	20 (16.1%)	30 (19.7%)	315,030 (10.9%)
>65	24 (19.4%)	19 (12.5%)	144,032 (5.0%)
*Females*	**66 (53.2%)**	**87 (57.2%)**	**1,542,467 (53.8%)**
18–44	21 (16.9%)	22 (14.5%)	979,030 (34.1%)
45–65	26 (21.0%)	41 (26.9%)	352,906 (12.3%)
>65	19 (15.3%)	24 (15.8%)	210,561 (7.3%)

^**+**^PAN = polyarteritis nodosa

^**++**^GPA = granulomatosis with polyangiitis.

**Table 2:** Event rates (per 10,000 patient years).

	PAN^+^	GPA^++^	Control
*AMIs* ^**∗**^ (%)			
18–44	9 (25.7%)	1 (2.6%)	8683 (0.47%)
45–65	3 (6.5%)	2 (2.8%)	29208 (4.37%)
>65	3 (7.0%)	2 (4.7%)	39319 (11.09%)

*CVAs* ^*∗∗*^ (%)			
18–44	0 (0%)	0 (0%)	1745 (0.09%)
45–65	6 (13.0%)	4 (5.6%)	11836 (1.77%)
>65	4 (9.3%)	1 (2.3%)	22434 (6.33%)

*AMI event rate* per 10,000 patient years (95% CI^*∗∗∗*^)			
*Males*			
18–44	268.1 (67.1–1070.2)	—	8.9 (8.7–9.2)
45–65	102.2 (14.4–725.8)	—	70.5 (69.6–71.5)
>65	171.2 (42.8–684.3)	139.5 (19.7–990.4)	181.1 (178.6–183.6)
*Females*			
18–44	172.3 (43.1–689.0)	71.5 (10.1–507.5)	1.9 (1.8–2.1)
45–65	—	—	22.9 (22.4–23.4)
>65	—	—	114.5 (112.9–116.1)

*CVA event rate* (per 10,000 patient years)			
*Males*			
18–44	—	—	1.2 (1.1–1.3)
45–65	381.4 (143.2–1016.3)	147.2 (36.8–588.5)	23.5 (23.0–24.1)
>65	173.9 (43.5–695.2)	—	99.2 (98.0–101.1)
*Females*			
18–44	—	—	0.9 (0.8–0.9)
45–65	182.8 (45.7–731.1)	112.4 (28.1–449.7)	13.2 (12.8–13.6)
>65	138.2 (19.5–981.2)	—	66.6 (65.4–67.8)

^+^PAN = polyarteritis nodosa

^++^GPA = granulomatosis with polyangiitis

^*∗*^AMI = acute myocardial infarction

^*∗∗*^CVA = cerebrovascular accident

^*∗∗∗*^CI = confidence interval.
